# Metabolic health in the Slovenian national cohort of kidney transplant patients 

**DOI:** 10.5414/CNP104S01

**Published:** 2025-11-28

**Authors:** Gregor Mlinšek, Anja Ponikvar Ležaić, Petra Finderle, Miha Arnol

**Affiliations:** 1 Department of Nephrology, University Medical Center Ljubljana,; 2 Faculty of Medicine, University of Ljubljana, and; 3 Clinical Institute for Clinical Chemistry and Biochemistry, University Medical Center Ljubljana, Ljubljana, Slovenia

**Keywords:** cardio-renal-metabolic syndrome, BMI, waist circumference, lipid profile, hs-CRP

## Abstract

Introduction: Metabolic health refers to the proper functioning and balance of metabolic processes in our bodies. The metabolism of carbohydrates, lipids, and proteins has a direct impact on the cardiovascular system. Recent advances in pharmacotherapy have introduced several drugs into clinical practice that can improve cardiometabolic health. Materials and methods: Between September 2023 and March 2024, we systematically collected cardiometabolic data from 800 kidney transplant patients (KTPs) during their routine outpatient visits. These included clinical data – office blood pressure, body weight and height, body mass index (BMI), and waist circumference (WC) – and laboratory data such as high-sensitivity C-reactive protein (hs-CRP), lipid profile, lipoprotein(a) (Lp(a)), glycated hemoglobin (HbA1c), serum urate, serum albumin, and proteinuria from spot urine samples. Patients who required treatment adjustment were selected. Results: Deviations from the desired values of individual components of the metabolic syndrome (blood pressure, WC, triglycerides, high-density lipoprotein cholesterol, and fasting glucose) were observed in 23 – 61.5% of patients. Elevated hs-CRP levels (5 – 10 mg/L), a known cardiovascular risk factor, were observed in 13.5% of patients. Lp(a) levels exceeded the upper normal limit (> 500 mg/L) in 17% of patients. Only a small proportion of patients with moderate to advanced kidney disease – 13% and 23.6%, respectively – had low-density lipoprotein cholesterol levels within the reference range. Conclusions: Between 23% and 61.5% of patients failed to meet target values for individual components of metabolic health. The largest deviation (61.5%) was observed in WC among women. WC and waist-to-height ratio are two simple and reliable parameters for assessing metabolic status.

## Introduction 

Metabolic health refers to the proper functioning and balance of metabolic processes in our bodies. The metabolism of carbohydrates, lipids, and proteins has a direct impact on the cardiovascular system. Cardio-renal-metabolic syndrome (CRMS), which involves a complex interplay of metabolic dysregulation, cardiovascular disease (CVD), kidney dysfunction, and diabetes risk factors, defines a patient’s overall metabolic health status. It encompasses obesity, insulin resistance, dyslipidemia, hyperuricemia, and hypertension, with obesity acting as a trigger for metabolic disturbances [1, 2]. 

CRMS is highly prevalent among kidney transplant patients (KTPs) and can adversely affect both post-transplant graft function and patient outcomes. Management of blood pressure, diabetes, albuminuria/proteinuria, dyslipidemia, and obesity is crucial to optimize care for patients after kidney transplantation. 

Nonpharmacological treatments, including diet modification, regular physical activity with psychological support, along with antihypertensive, lipid-lowering, and antidiabetic drugs, are used to address the individual components of metabolic syndrome. 

Recent advances in pharmacotherapy have introduced drugs such as sodium-glucose co-transporter 2 (SGLT2) inhibitors (empagliflozin and dapagliflozin), glucagon-like peptide-1 (GLP-1) receptor agonist semaglutide, the non-steroidal mineralocorticoid receptor (MR) antagonist finerenone, and proprotein convertase subtilisin/kexin type 9 (PCSK9) inhibitors (both monoclonal antibodies and inclisiran) into regular clinical practice. Although large-scale randomized controlled trials in KTPs are lacking, smaller studies indicate that SGLT2 inhibitors and GLP-1 receptor agonists are both feasible and safe in this patient population. New, effective lipoprotein(a) (Lp(a))-lowering drugs are currently in phase III clinical trials to determine whether reducing Lp(a) leads to meaningful cardiovascular benefits in the general population. 

To identify patients with uncontrolled individual cardiometabolic parameters and update their therapy, we systematically screened the entire Slovenian national cohort of KTPs with functioning grafts. 

## Materials and methods 

From September 2023 to March 2024, we collected cardiometabolic health parameters for each KTP during their regular visit to our outpatient clinic. These included clinical data (office blood pressure, body weight and height, body mass index (BMI), waist circumference (WC)) and laboratory data such as high-sensitivity C-reactive protein (hs-CRP), lipid profile, Lp(a), glycated hemoglobin (HbA1c), serum urate, serum albumin, and proteinuria in spot urine samples. Relative atherosclerotic risk (RTA) was calculated for each individual patient. Data on albuminuria (urine albumin-to-creatinine ratio (UACR)) were recorded in the second half of 2024. Basic statistical analysis was performed using Microsoft Excel. 

The study was approved by the National Medical Ethics Committee of the Republic of Slovenia (approval number: 0120-482/2023/5), and all patients provided written informed consent for their voluntary participation. 

## Results 

At the time of analysis, the Slovenian cohort of KTPs consisted of 800 prevalent patients with functioning grafts. 62% of them were men. Of the patients, 390 (48.8%) had their kidney graft for less than 10 years, 261 (32.6%) for 10 – 20 years, 128 (16%) for 20 – 30 years, 20 (2.5%) for 30 – 40 years, and 1 patient had a graft for over 40 years. Until 2012, our immunosuppressive protocol for patients with low immunological risk consisted of basiliximab, methylprednisolone, mycophenolate, and cyclosporine. Since 2012, tacrolimus has been used instead of cyclosporine. 

Immunosuppression data were collected for 791 patients (98.8% completeness). Dual immunosuppressive therapy was most common (67.3%), followed by triple therapy (28.7%) and monotherapy (4.0%). Steroids were used in 33.5% of patients – primarily methylprednisolone; only 4 received prednisolone. An additional 7.2% were switched to hydrocortisone after steroid discontinuation. Calcineurin inhibitors were prescribed to 98.7% of patients: tacrolimus in 75% (83.4% on the extended-release formulation) and cyclosporine in 25%. Six patients received belatacept instead of a calcineurin inhibitor. Antiproliferative agents were given to 91.4% of patients: 83.2% received mycophenolate (either mofetil or enteric-coated sodium), 12.6% everolimus, and 4.2% azathioprine. 

Estimated glomerular filtration rate (eGFR) ranged from 6 to ≥ 90 mL/min/1.73m^2^, with a median value of 57 (interquartile range (IQR) 42 – 72) mL/min/1.73m^2^. 65 (8.1%) patients were in stage 1 chronic kidney disease (CKD), 306 (38.3%) in stage 2 CKD, 344 (43%) in stage 3 CKD, 65 (8.1%) in stage 4 CKD, and 20 (2.5%) patients in stage 5 CKD. The median urinary protein-creatinine ratio (UPCR) was 13.2 g/mol (IQR 4.2 – 39), and the median UACR was 5.1 g/mol (IQR 1.3 – 18.9). 

### BMI and WC 

Data completeness for BMI and WC was 95%. We analyzed 474 male and 286 female patients with available data. BMI in the male subgroup ranged from 14.8 to 49.9 kg/m^2^, with a median value of 25.7 (IQR 23.3 – 28.7) kg/m^2^. BMI in the female subgroup ranged from 16.3 to 56.4 kg/m^2^, with a median value of 25.2 (IQR 22.4 – 28.9) kg/m^2^. 

The majority of patients had an elevated BMI, with 38.8% classified as overweight (BMI 25 – 29.9 kg/m^2^) and 16.7% as obese (BMI > 30 kg/m^2^). Only 42.6% had a normal BMI (18.5 – 24.9 kg/m^2^). Among the obese patients, 23 were classified as having class 2 obesity (BMI 35.0 – 39.9 kg/m^2^), and 4 as class 3 obesity (BMI > 40 kg/m^2^). 14 (1.8%) patients (6 men and 8 women) had a low BMI (< 18.5 kg/m^2^). 

WC ranged from 56.5 to 139 cm, with a median value of 92 (IQR 84 – 101) cm in the female subgroup and from 66 to 152 cm, with a median value of 99 (IQR 92 – 106) cm in the male subgroup. Combined assessment of BMI and WC enabled patient risk stratification, as presented in Table 1. 

Waist-to-height ratio (WHtR) in the male subcohort ranged from 0.35 to 0.87, with a median value of 0.56 (IQR 0.52 – 0.61). 70 patients (14.7%) had a WHtR < 0.5, 249 patients (52.5%) had a WHtR ≥ 0.5 to < 0.6, and 155 patients (32.7%) had a WHtR ≥ 0.6. In the female subcohort, WHtR ranged from 0.35 to 0.85, with a median value of 0.56 (IQR 0.52 – 0.62). 53 patients (18.5%) had a WHtR < 0.5, 134 patients (46.8%) had a WHtR ≥ 0.5 to < 0.6, and 99 patients (34.6%) had a WHtR ≥ 0.6. 

### Laboratory parameters 

The fasting lipid profile, including total cholesterol, low-density lipoprotein (LDL) cholesterol, high-density lipoprotein (HDL) cholesterol, triglycerides, and Lp(a), is presented in Table 2. Eleven (13%) of the 85 patients in CKD stages 4 and 5 had LDL cholesterol levels below 1.4 mmol/L, while 81 (23.5%) of the 344 patients in CKD stage 3 had LDL cholesterol levels below 1.8 mmol/L. A total of 564 (70.5%) patients had Lp(a) levels below 300 mg/L (the lower normal limit), with 299 (53%) of them falling below the lower limit of detection (< 87.5 mg/L). Lp(a) levels were greater than 500 mg/L (the upper normal limit) in 136 (17%) patients, and in 2 patients, Lp(a) levels exceeded 1,800 mg/L. 

The ranges, median values, and IQRs for blood pressure, hs-CRP, fasting blood glucose, HbA1c, serum albumin, serum urate, RTA, and albuminuria are presented in Table 3. Data completeness was high across all categories. 

## Discussion 

The key findings of our study indicate that, with the exception of serum albumin concentration, there is significant therapeutic potential to improve the cardiometabolic health of our patients across all observed clinical and laboratory parameters. Although serum albumin is not typically classified as a classic cardiometabolic parameter – such as blood pressure, lipid levels, glucose, or WC – it is an important biomarker with cardiometabolic relevance. The greatest deviation from target values was observed in our patients’ excessive body weight. This is more significant than it may appear, as obesity is not merely a risk factor in itself; rather, it is considered a central driver of numerous downstream risks, including the development of type 2 diabetes, hypertension, dyslipidemia, increased systemic inflammation, oxidative stress, and endothelial dysfunction. Consequently, it significantly amplifies the risk of heart failure, coronary artery disease, and stroke. 

### BMI, WC, and WHtR 

**Figure 1:**
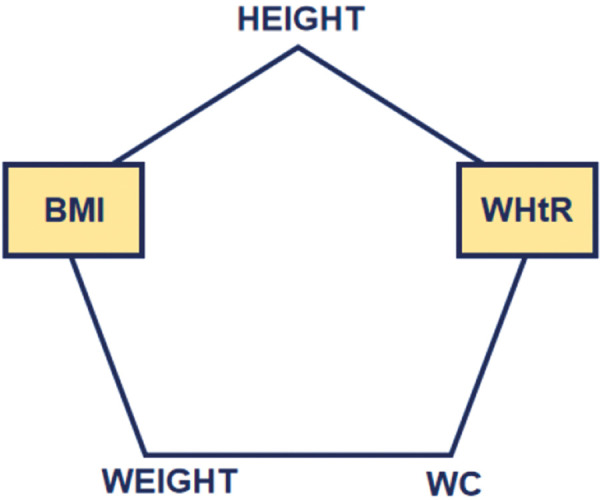
Commonly used anthropometric indicators related to cardiometabolic risk. BMI = body mass index; WC = waist circumference; WHtR = waist-to-height ratio.

There are several anthropometric indicators related to cardiometabolic risk, such as BMI, WC, WHtR, body fat percentage, and the conicity index [3]. Several of these are depicted in Figure 1. 

WC is a key indicator of visceral fat, the type of fat that surrounds internal organs such as the heart, kidneys, liver, digestive organs, and pancreas. Visceral fat is more metabolically active than subcutaneous fat and contains a higher concentration of immune cells, particularly macrophages, that can produce inflammatory cytokines. However, not all visceral fat is equally harmful; in some individuals, especially those who are metabolically healthy, it may not trigger a strong inflammatory response. This variability may be influenced by genetics, diet, gut microbiome, physical activity, and the size versus number of fat cells. Numerous large studies have shown a strong association between abdominal obesity and an increased risk of hypertension, dyslipidemia, and type 2 diabetes – all of which independently elevate the risk of CVD and stroke [4, 5]. Therefore, combined measures of BMI and WC may offer a more comprehensive assessment of CVD risk [6]. Table 1 shows the combination of BMI and WC in our KTPs, with 163 (34.4%) male patients and 128 (44.7%) female patients in the high cardiometabolic risk category. 

WHtR may be an even simpler and more predictive indicator of the “early health risks” associated with central obesity. Meta-analyses of data in adults of all ages have supported the superiority of WHtR over the use of BMI with WC in predicting early health risks [7]. A healthy WC, measured at the navel, should be less than half the body height. Therefore, WHtR < 0.5 indicates no increased risk, WHtR ≥ 0.5 to < 0.6 indicates “increased risk”, and WHtR ≥ 0.6 indicates “very high risk”. The percentage of very high cardiometabolic risk patients as assessed by WHtR, compared to the high risk group as derived from the BMI-WC measurement was similar in male patients (32.7% and 34.4%) and slightly lower in female patients (34.6% and 44.8%). 

Patients with high cardiometabolic risk are candidates for therapeutic interventions, including dietary counseling, physical activity, hormonal testing, psychological support, and pharmacotherapy with SGLT2 inhibitors and GLP-1/GIP receptor agonists, aiming at gradual fat loss while preserving as much muscle mass as possible. Although large-scale randomized controlled trials in KTPs are lacking, a 2024 systematic review and meta-analysis encompassing 18 studies (17 observational and 1 RCT) involving diabetic KTPs found that both SGLT2 inhibitors and GLP-1 receptor agonists were associated with improved glycemic control and weight reduction, without significant adverse effects on kidney function or blood pressure [8, 9]. Undernourished patients, on the other hand, need dietary counseling, oral nutritional supplements, and a physical exercise program to reverse sarcopenia and achieve a healthy body fat percentage [10]. 

Serum albumin was the parameter where we found the greatest agreement between actual and desired values. In almost all patients (99.2%), the measured value was within the normal range. This suggests that our patients were generally not undernourished, which is consistent with the BMI, WC, and WHtR findings. Only 14 patients (8 women and 6 men) were considered undernourished according to BMI < 18.5, which is consistent with only 5 (0.6%) patients having serum albumin concentrations < 32 g/L. 

### Metabolic syndrome 

There are several definitions of metabolic syndrome. According to the International Diabetes Federation (IDF) and the American Heart Association/National Heart, Lung, and Blood Institute (AHA/NHLBI), metabolic syndrome is defined as the combination of any 3 of the following: elevated WC in men > 102 cm, in women > 88 cm; triglycerides ≥ 1.7 mmol/L; HDL cholesterol in men < 1.03 mmol/L, in women < 1.29 mmol/L; blood pressure ≥ 130/85 mmHg; and/or fasting glucose > 6.1 mmol/L [11]. We need to stress that there are no transplant-specific universal targets, but general cardiovascular risk guidelines are often applied, specifically for WC in men < 102 cm and in women < 88 cm. These thresholds come from WHO and NIH guidelines and are often used in transplant follow-up to assess metabolic health and guide lifestyle interventions. 

Data presented in Table 4 show that all individual components of metabolic syndrome deviate from the desired values in roughly 1/5 to 1/3 of patients, both in the male and female subgroups. An exception in the female subgroup was the high percentage (61.5%) of large WC, and in the male subgroup, a high percentage (51%) of low HDL cholesterol concentration. 

There is a strong and well-established correlation between WC and high-sensitivity C-reactive protein (hs-CRP) levels [12]. Although some individuals store fat viscerally, they may still maintain good metabolic health – for a time. In our male patient cohort, 138 out of 172 men (80.2%) with a WC greater than 102 cm had hs-CRP levels below 5 mg/L, suggesting that they may still be metabolically healthy. Similarly, 135 out of 175 women (77.1%) with a WC over 88 cm had hs-CRP levels below 5 mg/L. While visceral fat is a significant health risk, it is not necessarily linked to systemic inflammation; however, the likelihood of inflammation increases with greater fat accumulation, especially when combined with poor lifestyle factors. 

Weight reduction is important in both groups with increased WC, those with and without elevated hs-CRP. However, it is particularly urgent in individuals with elevated hs-CRP, as inflammation significantly increases the risk of metabolic and cardiovascular complications, even when other markers appear normal. 

Weight reduction and improvement of other metabolic syndrome parameters can be achieved through non-pharmacological approaches such as dietary modification and increased physical activity – both aerobic and resistance training – as well as pharmacological treatments. These may include antihypertensive, antidiabetic, and lipid-lowering medications, along with newer options for obesity management such as SGLT2 inhibitors and/or GLP-1/GIP receptor agonists [8, 9, 10]. 

### Lipid profile 

KTPs with an eGFR < 30 mL/min/1.73m^2^ should have LDL cholesterol levels below 1.4 mmol/L [12]. However, in our cohort, only 11 (13%) of 85 patients in CKD stages 4 and 5 met this requirement, indicating that the majority of patients with advanced CKD had inappropriately high LDL cholesterol. Only 81 (23.6%) of 344 patients with CKD stage 3 had LDL cholesterol levels < 1.8 mmol/L. Although not all patients in the latter group require secondary cardiovascular prevention to achieve this LDL concentration, our data suggest there is considerable room for improvement in hypolipidemic therapy with statins, ezetimibe, and PCSK9 inhibitors for some patients. 

Lp(a) increases the likelihood of heart attack, stroke, and aortic stenosis, especially in cases of familial hypercholesterolemia and coronary heart disease [13]. Normal Lp(a) is considered to be below 300 mg/L, while elevated levels are considered to be above 500 mg/L. Mendelian randomization studies employing polygenic risk scores suggest that extremely elevated Lp(a) levels > 1,800 mg/L are a risk factor for atherosclerotic cardiovascular disease, comparable to heterozygous familial hypercholesterolemia [14]. 

In our cohort, 136 (17%) patients had Lp(a) levels above 500 mg/L, 34 of whom had Lp(a) > 1,000 mg/L, and 2 had levels > 1,800 mg/L. Lp(a) levels are largely genetically determined, but both cyclosporine and tacrolimus can lead to an increase in Lp(a). Among the 136 patients with Lp(a) > 500 mg/L, 92 were treated with tacrolimus, 42 with cyclosporine, and 2 with belatacept. Patients with Lp(a) > 1,000 mg/L are, according to our National Health Insurance Institute, eligible for treatment with PCSK9 inhibitors, which can reduce Lp(a) by about 20 – 30% on average. Whether Lp(a) reduction translates into significant improvements in cardiovascular outcomes, both in the general population and specifically in kidney transplanted patients, is still being investigated. Currently, we do not routinely treat patients with PCSK9 inhibitors solely for elevated Lp(a). 

### hs-CRP and RTA 

In 80 patients, hs-CRP levels were above 10 mg/L. As these elevations were attributed to acute infections or transient inflammatory conditions, they were not interpreted as markers of future cardiovascular risk. However, since our study had a cross-sectional design, we did not follow these patients longitudinally and cannot exclude the possibility that some may have had persistently elevated hs-CRP levels even after resolution of the presumed acute condition. The remaining 704 hs-CRP values ranged from 0.2 to 10 mg/L, with a median value of 1.5 (IQR 0.8 – 3.3) mg/L. Literature data show that KTPs with a CRP above 5 mg/L had increased mortality compared to patients with levels below that threshold [15, 16]. In the JUPITER trial, apparently healthy individuals without hyperlipidemia but with elevated hs-CRP levels had a significant reduction in the incidence of major cardiovascular events when treated with rosuvastatin [17]. In our cohort, 195 patients with hs-CRP levels between 5 and 10 mg/L would possibly need additional lifestyle modifications, statin therapy, and, in the case of secondary prevention, acetylsalicylic acid. 

RTA is an atherosclerotic risk model based on a combination of hs-CRP and the ratio of total cholesterol to HDL cholesterol. Both parameters together predict atherosclerotic risk better than either parameter individually [18]. In this model, hs-CRP values above 15 mg/L (the 99^th^ percentile in the general population) were presumed to reflect acute or non-specific inflammation (e.g., due to infection), rather than the low-grade chronic inflammation associated with metabolic syndrome or atherosclerosis. Therefore, RTA was not calculated for those patients. Data completeness for RTA was somewhat lower (91.9%) compared to other parameters. The median RTA value in our KTPs was 2.2 (IQR 1.4 – 3.5), with a maximum relative risk of 8.7 (observed in 14 patients). In addition to using generally accepted tools for assessing cardiovascular risk, which often omit or underestimate the risk arising from CKD, relative atherosclerotic risk can serve as an additional criterion when deciding on the initiation and intensity of cardiovascular prevention. 

### Albuminuria 

Leakage of albumin through the glomerular vessels causes albuminuria, while leakage through the vascular endothelium elsewhere results in leakage into the vascular interstitium, where albumin induces inflammation and atherosclerosis. Albuminuria is, therefore, an important early predictor of both kidney and heart disease outcomes. The risk of cardiovascular death increases significantly once the UACR exceeds 1 g/mol [19]. In our cohort, 58% of patients had albuminuria classified as group A2 or A3. Treatment with ACE inhibitors or sartans, SGLT2 inhibitors, GLP-1 receptor agonists, and finerenone should be instituted in eligible individuals. 

### Hyperuricemia 

Serum uric acid (SUA) plays a role in increasing cardiovascular risk in KTPs, primarily through mechanisms like endothelial dysfunction, inflammation, hypertension, and atherosclerosis. Some immunosuppressive medications, particularly calcineurin inhibitors (e.g., tacrolimus), can either increase urate production or decrease its excretion, leading to elevated urate levels. The median SUA in our patients was 404 (IQR 343 – 472) µmol/L. One study reported that KTPs with post-transplant SUA > 404 µmol/L had a significantly higher risk of developing congestive heart failure [20]. Although allopurinol has been shown to lower uric acid levels and potentially improve cardiovascular risk markers like endothelial dysfunction and inflammation in kidney-transplanted patients, there is still insufficient evidence to conclusively determine its effect on reducing major cardiovascular events in this population. 

### Fasting glucose and HbA1c 

The prevalence of diabetes in KTPs can range from 30 to 70%, considering both pre-existing diabetes (30 – 40%) and the development of new-onset diabetes after transplantation (up to 30%) [21]. Based on 114 patients with HbA1c > 6.5%, the prevalence of diabetes in our cohort was at least 14.4%. However, this value is likely underestimated, as some patients with well-controlled diabetes have HbA1c levels within the normal range. Additionally, 100 patients (12.6%) had fasting glucose levels > 7 mmol/L, suggesting a similar prevalence of diabetes. 

## Conclusion 

Systematic analysis of cardiometabolic health parameters can help identify patients who require therapeutic optimization. We found deviations in individual parameters in up to 61.5% of our KTPs. Two simple and reliable health parameters, WC and WHtR, were particularly useful for this assessment. Although large-scale randomized controlled trials on pharmacological and non-pharmacological treatments in KTPs are lacking, numerous smaller studies suggest that interventions such as weight reduction in obese patients and the use of SGLT2 inhibitors and GLP-1 receptor agonists are associated with improved glycemic control, weight loss, and reduction in UPCR ratio in this specific patient population, supporting their current use. 

## Acknowledgment 

The authors would like to thank the nurses of the Kidney Transplant Center for their consistent implementation of the protocol and Martina Milošič, registered nurse, for her help with data collection. 

## Authors’ contributions 

Conceptualization, G.M.; methodology, G.M.; acquisition of patient data, G.M., A.P.L., and P.F.; validation, G.M.; formal analysis, G.M.; writing – original draft preparation, G.M.; writing – review and editing, M.A., P.F., and A.P.L. All authors have read and approved the final version of the manuscript. 

## Funding 

This research was funded by the Public Research Agency of the Republic of Slovenia, program number ARRS P3-0323. 

## Conflict of interest 

The authors declare no conflict of interest. 

**Table 1.** Table 1. Cardiometabolic risk stratification based on BMI and waist circumference.

Parameter		Female (286 pts)			Male (474 pts)		
Waist circumference		Low	High	Very high	Low	High	Very high
		< 80 cm	80 – 88 cm	> 88 cm	< 94 cm	94 – 102 cm	> 102 cm
BMI	< 18.5 underweight	8 (2.8%)	0	0	6 (1.3%)	0	0
	18.5 – 24.9 normal	31 (10.8%)	52 (18.2%)	47 (16.4%)	114 (24%)	71 (15%)	9 (1.9%)
	> 25 overweight and obese	0	20 (7%)	128 (44.8%)	22 (4.6%)	89 (18.8%)	163 (34.4%)
Low risk	Medium risk	High risk	Risk not graded

BMI = body mass index.

**Table 2.** Fasting lipid profile, including total cholesterol, HDL-C, LDL-C, triglycerides, and Lp(a).

	Total cholesterol (mmol/L)	HDL-C (mmol/L)	LDL-C (mmol/L)	Triglycerides (mmol/L)	Lp(a) (mg/L)
Data completeness (%)	99.1	99.1	98.7	98.6	97.6
Minimal	1.9	0.4	0.7	0.2	< 87.5^#^
Maximal	8.5	5.6	5.7	14.4	2,630
Median	4.3	1.1	2.5	1.3	107
IQR	3.7 – 4.9	0.9 – 1.4	2-3.1	1-1.8	87.5 – 330

^#^Below the lower limit of detection. HDL-C = high-density lipoprotein cholesterol; LDL-C = low-density lipoprotein cholesterol; Lp(a) = lipoprotein(a).

**Table 3.** Blood pressure, hs-CRP, fasting blood glucose, HbA1c, serum albumin, serum urate, RTA, and albuminuria.

	SBP (mmHg)	DBP (mmHg)	hs-CRP (mg/L)	FBG (mM)	HbA1c (%)	SA (g/L)	SU (μmol/L)	RTA	UACR (g/mol)
Data completeness (%)	95.7	95.7	97.7	95.7	98.5	99.1	99.5	91.9	94.6
Minimal	80	42	0.2	1.3	3.4	28	155	0.8	0.2
Maximal	216	135	10	16.2	11	58	760	8.7	1,767.5
Median	135	76	1.5	5.3	5.6	45	404	2.2	5.1
IQR	123 – 147	67 – 84	0.8 – 3.3	4.8 – 6.1	5.3 – 6.0	42 – 46	343 – 472	1.4 – 3.5	1.3 – 18.9

SBP = systolic blood pressure; DBP = diastolic blood pressure; hs-CRP = high sensitivity C-reactive protein; HbA1c = glycated hemoglobin; FBG = fasting blood glucose; SA = serum albumin; SU = serum urate; RTA = relative atherosclerotic risk; UACR = urine albumin-creatinine ratio; mM = millimoles per liter (mmol/L).

**Table 4.** Metabolic syndrome parameters for male and female patients in our national cohort of kidney-transplanted patients.

	Reference^#^	Female (286)	Male (474)
Blood pressure	≥ 130/85 mmHg	68 (23.7%)	94 (19.8%)
Waist circumference	> 102 cm (male), > 88 cm (female)	176 (61.5%)	172 (36.2%)
Triglycerides	≥ 1.7 mM	97 (33.9%)	153 (32.2%)
HDL-C	< 1.03 mM	66 (23%)	242 (51%)
Fasting glucose	> 6.1 mM	66 (23%)	120 (25.3%)

^#^Definition of metabolic syndrome endorsed by the International Diabetes Federation (IDF), the American Heart Association (AHA), and the National Heart, Lung, and Blood Institute (NHLBI). HDL-C = high-density lipoprotein cholesterol; mM = millimoles per liter (mmol/L).
